# Exploring the Ecological Performance of China’s Tourism Industry: A Three-Stage Undesirable SBM-DEA Approach with Carbon Footprint

**DOI:** 10.3390/ijerph192215367

**Published:** 2022-11-21

**Authors:** Yufeng Chen, Zhitao Zhu, Lin Zhuang

**Affiliations:** 1 School of Economic and Management, Zhejiang Normal University, Jinhua 321004, China; 2 School of Statistics and Mathematics, Zhejiang Gongshang University, Hangzhou 310018, China; 3 School of Economics, Zhejiang Gongshang University, Hangzhou 310018, China

**Keywords:** tourism industry, eco-efficiency, carbon footprint, ecological performance

## Abstract

The environmental impact of carbon emissions and the carbon footprint from tourism activities are significant for promoting low-carbon development in the tourism industry. This paper employed a bottom-up approach to estimate the carbon footprint and energy consumption of China’s tourism industry. Then, the three-stage undesirable SBM-DEA model was employed to evaluate and decompose the eco-efficiency of China’s provincial tourism industry from 2008 to 2017. The results showed that the eco-efficiency of most provinces has experienced a slight increase during the past ten years, while management inefficiency within the tourism industry has been the main restriction of the utilization of tourism resources in most regions. The decomposition and quadrant analysis indicated that scale efficiency was the direct driver of the poor ecological performance in Northeast China, while technical efficiency dominated the tourism eco-efficiency in South-Central China. These two issues have together led to the poor utilization of the rich tourism resources and the natural environment in Southwest China. On the basis of these discussions, differentiated policy implications towards different kinds of regions were provided to promote low-carbon development and to realize the potential of tourism resources in China’s tourism industry.

## 1. Introduction

Tourism, as one of the most promising industries in modern economics, shows strong abilities for low-carbon sustainable development and risk resistance. According to the World Travel and Tourism Council (WTTC), tourism contributed 10.3% (USD 9630 billion) to global GDP and 10% (333 million jobs) to global employment in 2019. Despite the continuous shock from COVID-19, the tourism sector still contributes 6.1% (USD 5812 billion) and 9.1% (289 million jobs) to global GDP and employment in 2021 (data come from Global Economic Impact Reports, World Travel and Tourism Council, https://wttc.org/research/economic-impact, accessed on 6 September 2022.). As the world’s largest developing country, China is an important tourism destination, and contributed 11.6% (USD 81,856.6 billion) to the total economy in 2019 and 4.6% (USD 814.3 billion) in 2021 (data come from Global Economic Impact Reports (China), World Travel and Tourism Council, https://wttc.org/research/economic-impact, accessed on 6 September 2022). As high-quality development has become the goal of China’s economic development, the Chinese government has paid more and more attention to the balance between economic development and environmental protection, and low-carbon development has become the consensus of policy makers. Under such a background, the tourism industry has naturally become an important engine of green development for its strong relationship with inclusive and green growth [1].

On the other hand, the tourism industry is always regarded as being more eco-friendly than other industries, leading to the situation that most of the tourism promotion policies put economic performance in first place and do not provide a clear expression of sustainability in tourism [2]. Although manufacturing is still the main contributor for carbon emission [3], this paradox has resulted in a sharp increase in fossil energy consumption caused by the booming tourism development, becoming an important incentive for climate change. The greenhouse gas emissions of China’s tourism industry also show a growing trend [4,5,6]. The damage to the ecological environment becomes an inescapable obstacle to the development of sustainable tourism. As an eco-friendly sector, the tourism industry needs to realize the coordinated development of economy, environment, and ecology. 

Hence, the assessment of tourism performance needs to combine economic, environmental, and ecological factors into one framework. Considering the resource consumption and carbon emission in each tourism activity, the evaluation of the tourism industry should not only include its economic performance but also its negative impacts on the environment. Eco-efficiency, defined as the utilization efficiency of ecological resources to obtain certain economic and environmental targets, is an important tool for assessing the low-carbon development of tourism. Usually, a higher eco-efficiency value means that fewer inputs are required to achieve the same economic and ecological goals or the same inputs can achieve higher economic and ecological goals. It can not only reflect the core connotation of the sustainable development theory but can also emphasize the harmonious integration of social and economic development and resource environmental protection. 

This paper employed the bottom-up approach to estimate the carbon footprint and energy consumption of China’s tourism industry. Then, the three-stage undesirable SBM-DEA model was employed to evaluate and decompose the eco-efficiency of China’s provincial tourism industry from 2008 to 2017. This paper contributes to current research on the following issues. Firstly, this paper combined the bottom-up method and the three-stage undesirable SBM-DEA framework to give a comprehensive analysis on the environmental and low-carbon performance of China’s tourism industry. Secondly, decomposition and quadrant analyses were employed to discover the driving force of the low-carbon performance, and differentiated policy implications towards different categories of regions were provided to promote the low-carbon performance and to realize the potential of the tourism resources of China’s tourism industry.

The remainder of this study is organized as follows. Section 2 reviews the literature on the eco-efficiency of the tourism industry and its measurement. Section 3 introduces the model and Section 4 describes the data and variables. Section 5 measures and discusses the results of the eco-efficiency of the tourism industry. Section 6 concludes the study and provides some policy implications for improving the eco-efficiency of the tourism industry.

## 2. Literature Review

Eco-efficiency was developed by the World Business Council for Sustainable Development (WBCSD) and now is a widely used indicator for measuring the environmental performance of an economic activity. It is generally recognized that eco-efficiency refers to the utilization efficiency of ecological resources to obtain certain economic or environmental output targets. The eco-efficiency of the tourism industry was clearly proposed by Gossling et al. (2005) and was believed to be an important indicator for guiding the sustainable development of tourism [6]. 

As the world’s largest emerging industry, the impact of tourism on the ecological environment has become one of the current hot issues. However, many scholars mainly focus on the operational efficiency and productivity of tourism enterprises, such as tourist hotels [7,8,9], travel agencies [10,11], tourist attractions [12], and tourist transportation [13]. With the intensification of global warming, some scholars are also beginning to pay attention to the impact of tourism on the environment. However, this stream of the literature mainly focuses on certain parts of tourism, including tour route products [14], tourism destinations [15,16], tourism waste [17], tourism traffic [18,19], and tourism carbon emissions [20]. For example, Sun (2016) investigated the dynamic relationship between tourism development, technological efficiency, and carbon emissions and found that technological innovation can hardly cover the sharp increase of tourism carbon emissions [21]. Song and Li (2019) analyzed the sustainable development efficiency of China’s tourism industry, with the consideration of governmental environment investment, to uncover the factors that have an influence on the efficiency of China’s tourism industry [22]. Another stream of the literature evaluates the sustainable development of tourism by composite indicator [23,24]. However, existing research has not provided an accurate measurement for the harmonious development of tourism, resources, and the environment.

Eco-efficiency assessment is a complicated and multidisciplinary task [25]. Many scholars have extended the measurement methods of eco-efficiency, including life-cycle assessment [26,27], material flow analyses [28,29], ecological footprints [30], and data envelopment analysis (DEA) [31]. Among them, the DEA model is one of the most widely used methods because of some of its special advantages in dealing with the issue of multi-inputs and multi-outputs [32,33,34]. Since Kuosmanen and Kortelainen (2005) first used the DEA method to analyze eco-efficiency [35], scholars have developed various DEA-based models to conduct environmental impact assessment and to evaluate eco-efficiency [36]. For instance, Rashidi and Farzipoor (2015) employed a non-radial DEA model to calculate and analyze the eco-efficiency of OECD countries [37]. The results show that the energy input is directly proportional to the non-expected output, that is, the more energy input, the more non-expected output. Zhang et al. (2008) developed a DEA-based model and measured the eco-efficiency of China’s industrial systems and found that economy development will promote the eco-performance of industrial sectors [38].

In view of the current literature, some gaps need to be further studied. Firstly, there is no unified definition of the concept of ecological efficiency. This paper combined the energy consumption and the carbon footprint into the multiple inputs and multiple outputs of the DEA framework to form a new perspective on measuring tourism ecological efficiency. Secondly, current research about the eco-efficiency of the tourism industry mainly focuses on the dynamics and regional differences, paying less concern to the driving force of the inefficiency. This paper decomposed the eco-efficiency into scale efficiency and technology efficiency to discuss the driving force in different regions and provided differentiated policy implications towards different categories of regions to promote the low-carbon performance and to realize the potential of tourism resources in China’s tourism industry.

## 3. Methodology, Data, and Variables

### 3.1. The Bottom-Up Approach

There were two methods for the measurement of carbon emissions and energy consumption in the tourism industry, namely the bottom-up and the up-bottom methods. The bottom-up method refers to the summation of carbon emissions and energy consumption from various sub-sectors in the tourism industry, while the up-bottom approach mainly relies on tourism satellite accounts that evaluate carbon emissions and energy consumption from the total level. Since China has not established a national- or regional-level greenhouse gas emission monitoring system, this paper employed the bottom-up method to measure the CO_2_ emissions and energy consumption of the tourism industry by dividing it into three sub-sectors (tourism transport, tourism accommodation, and tourism activities), then estimated energy consumption and carbon emissions for each sub-sector [4]^.^ According to Shi et al. (2011), the specific method is as follows [39].

(1) $$TC_{tj}=TC_{Ttj}+TC_{Ltj}+TC_{Atj}$$

$TC_{tj}$ represents carbon emissions and energy consumption of the tourism industry; $TC_{Ttj},TC_{Ltj},TC_{Atj}$ represent the carbon emissions and energy consumption of tourism transportation, tourism accommodation, and tourism activities, respectively.

Specifically, $TC_{Ttj}$ can be obtained by Equation (2):

(2) $$TC_{Ttj}=\sum_{i=1}^{n}Q_{itj}\cdot f_{i}\cdot \alpha_{i}$$

where $Q_{itj}$ is the passenger turnover in the mode *i* of vehicles (including highway, railway, aviation, water transport) in province *j* of year *t*. $f_{i}$ represents the proportion of tourists in the passenger traffic of mode *i* of vehicles. $\alpha_{i}$ represents the carbon emission and the energy consumption coefficient factor for the transportation mode *i* (Table 1). 

$TC_{Ltj}$ could be obtained by Equation (3):

(3) $$TC_{Ltj}=N_{tj}\cdot P_{t}\cdot \beta$$

where $N_{tj}$ is the number of beds in a star hotel in province *j* of year *t*, $P_{t}$ represents average room-occupancy rate, and β represents carbon emissions and energy consumption per bed per night. According to the study of Shi et al. (2011), the value of $P_{t}$ and β was selected as 2.458 g per visitor per night and 155 MJ per visitor per night [39].

$TC_{Atj}$ can be obtained by Equation (4):

(4) $$TC_{Atj}=\sum_{k=1}^{n}P_{ktj}\cdot \eta_{k}$$

where $P_{ktj}$ represents the number of people participating in mode *k* of tourism activities and $\eta_{k}$ is the carbon emission or the energy coefficient factor for each tourism activity. According to the current research of Shi et al. (2011), the values of these parameters are shown in Table 2 [39].

### 3.2. Three-Stage DEA Model

**Figure 1:**
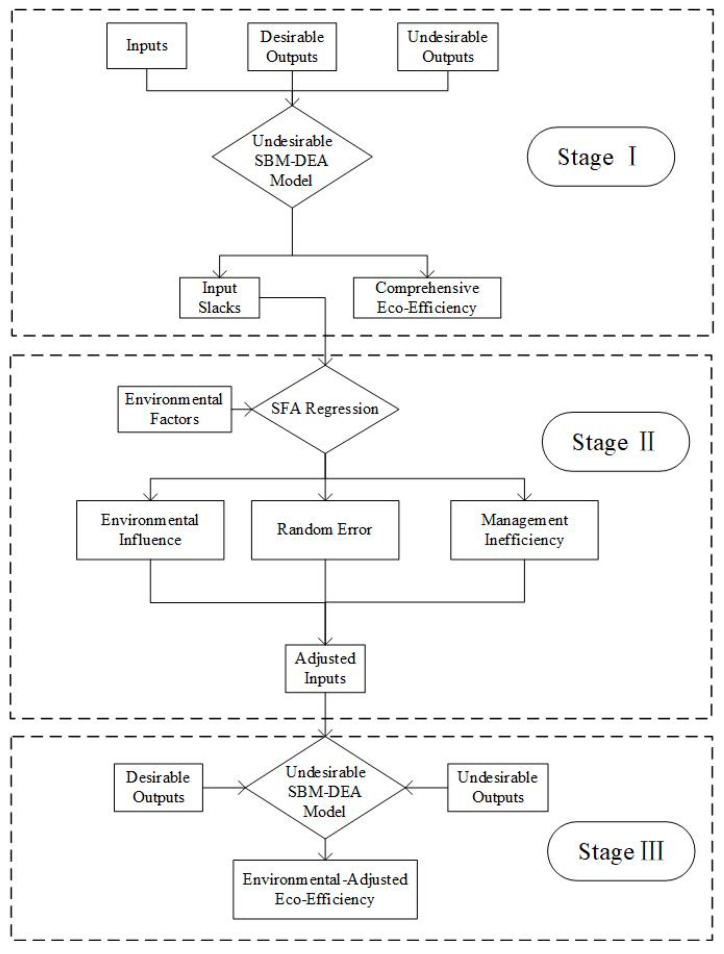
Methodological framework of the evaluation of eco-efficiency of the tourism industry.

The original DEA model, first put forward by Charnes and Cooper (1984), was widely employed to evaluate the relative effectiveness of different decision-making units (DMUs) [40]. However, the traditional DEA model does not eliminate the influence of management inefficiency, environmental effects, and statistical noise in the efficiency evaluation of DUMs. Fried et al. (2002) proposed a new efficiency evaluation model, the three-stage DEA model, which can effectively eliminate the influence of environmental factors and random errors, thus reflecting the actual efficiency [41]. This paper combined the three-stage DEA model and the undesirable SBM model to evaluate the eco-efficiency of China’s tourism industry; the methodological framework is illustrated in Figure 1.

#### 3.2.1. Stage Ⅰ: The Undesirable SBM Model

The SBM model, first put forward by Tone (2001), can consider both input and output slack variables [42]. However, the SBM model cannot deal with undesirable outputs. Dyckho and Allen (2001) counted the undesirable output as the input to calculate the eco-efficiency, but this may lead to a decrease in the efficiency value [43]. To address this problem, Tone (2003) introduced the undesirable output based on the traditional SBM model and proposed the undesirable SBM model [44]. 

According to Tone’s method, this paper assumed there are *n* DMUs and each DMU $\left(j=1,2,\cdots ,30\right)$ consumes types i of inputs $x_{ij}\left(i=1,2,3\right)$, which represent labor, capital, and energy, respectively. $y^{g}$ represents tourism revenue and $y^{b}$ represents carbon emission. Hence, the production possibility set P is:

(5) $$P=\left\{\left(x,y^{g},y^{b}\right),x\geq \lambda x,y^{g}\geq \lambda y^{g},y^{b}\geq \lambda y^{b},\lambda \geq 0\right\}$$

The SBM undesirable model based on variable returns to scale (VRS) is expressed as below:

(6) $$\begin{array}{l} p^{*}=\min\frac{1-\frac{1}{m}\sum_{i=1}^{m}\frac{s_{i}^{-}}{x_{i0}}}{1+\frac{1}{s_{1}+s_{2}}\left[\sum_{r=1}^{s_{1}}\frac{s_{r}^{g}}{y_{r0}^{g}}+\sum_{r=1}^{s_{2}}\frac{s_{r}^{b}}{y_{r0}^{b}}\right]}\\ s.t.\left\{\begin{array}{l} x_{0}=X\lambda +s\\ y_{0}=Y^{g}\lambda +s^{g}\\ y_{0}=Y^{b}\lambda +s^{b}\\ \lambda \geq 0,s^{-}\geq 0,s^{g}\geq 0,s^{b}\geq 0 \end{array}\right. \end{array}$$

where $s_{i}^{-}$, $s_{r}^{g}$, and $s_{r}^{b}$ represent the slacks of inputs, desirable outputs, and undesirable outputs, respectively. λ is the weight vector and $P^{*}\in \left[0,1\right]$ represents the eco-efficiency of tourism industry, which is strictly decreasing about $s_{i}^{-},s_{r}^{g},s_{r}^{b}$. One DMU is effective only when $P^{*}=1$ and $s_{i}^{-}=0,s_{r}^{g}=0,s_{r}^{b}=0$ and represents inefficiency when $P^{*}<1$ or $s_{i}^{-}\neq 0,s_{r}^{g}\neq 0,s_{r}^{b}\neq 0$.

#### 3.2.2. Stage Ⅱ: Stochastic Frontier Analysis 

The eco-efficiency value in Stage Ⅰ cannot separate the impacts of environmental factors, stochastic errors, and internal management factors on efficiency values. Therefore, an SFA model (Equation (7)) was established to eliminate the environmental effect and random noises, so that the slack of inputs only caused by management inefficiency could be obtained.

(7) $$S_{ik}=f^{i}\left(z_{k};\beta^{i}\right)+v_{ik}+u_{ik},i=1,2,\cdots ,m,k=1,2,\cdots ,n$$

In Equation (7), $S_{ik}$ represents the slack matrix for input k;$z_{k}=\left(z_{1k},z_{2k},\cdots ,z_{pk}\right)$ represents an external environment variable; $\beta^{i}$ is the parameter to be estimated of the external environment variable; $f^{i}\left(z_{k},\beta^{i}\right)$ represents the impact of external environment variables on the input slack $s_{ik}$;$v_{ik}+u_{ik}$ is the hybrid error; $v_{ik}\sim N\left(0,\sigma_{v}^{2}\right)$ refers to the stochastic error term; $u_{ik}\sim N\left(0,\sigma_{u}^{2}\right)$ denotes the management inefficiency; $\gamma =\sigma_{ui}^{2}/\left(\sigma_{ui}^{2}+\sigma_{vi}^{2}\right)$ represents the proportion of technical inefficiency variance in the total variance. When γ tends to 0, the influence of the random error term is dominant. When γ tends to 1, the influence of environmental factors on management inefficiency is dominant. Based on the optimal DMU and its input, each decision-making unit is adjusted according to Equation (8).

(8) $$\begin{array}{l} x_{ik}^{*}=x_{ik}+\left[\max_{k}\left\{z_{k}\beta^{i}\right\}-z_{k}\beta^{i}\right]+\left[\max_{k}\left\{v_{ik}\right\}-v_{ik}\right]\\ \left(i=1,2,\cdots ,m,k=1,2,\cdots ,n\right) \end{array}$$

where $x_{ik}^{*}$ is the adjusted inputs, $\left[\max_{k}\left\{z_{k}\beta^{i}\right\}-z_{k}\beta^{i}\right]$ represents the adjustment for external environmental factors and indicates that all DMUs were facing the same external environment, $\left[\max_{k}\left\{v_{ik}\right\}-v_{ik}\right]$ represents the adjustment of stochastic error and indicates that all DMUs are under the same level of luck (random noises). Equation (8) shows that all inputs will be adjusted within the same environmental factors and luck (random noises). If the adjusted inputs are lower than the initial inputs, these DMUs will have a higher efficiency value (under the same level of outputs). With this approach, the interference of external environment and random error will be unified to investigate the inherent management factors that influence the efficiency value of each DMU.

#### 3.2.3. Stage Ⅲ: Adjusted Undesirable SBM Model

The adjusted inputs and the original outputs are re-substituted into the SBM model to recalculate the efficiency values of each DMU. The result can more accurately reflect the eco-efficiency of the tourism industry because the influence of external factors was eliminated in Stage Ⅱ so that the efficiency value that only reflects the management level of tourism industry can be obtained. At the same time, through the estimated coefficient in Stage Ⅱ, the influence of external environmental factors on eco-efficiency could be discussed indirectly by examining how the environmental variables affect the input slacks.

### 3.3. Data and Variables

This paper selected the dataset of China’s province tourism industry between 2008 and 2017 (except Tibet, Hong Kong, Macao, Taiwan), which are mainly collected from the *China Statistical Yearbook*, the *China Tourism Statistics Yearbook*, the *China Transportation Yearbook*, China’s provincial statistical yearbooks, *the Sample Survey of National Tourism Bureau*, *and China’s provincial Statistical bulletin on national economic and social development*. According to the relevant literature [16,38,45] (Yin et al., 2014; Peng et al., 2017; Zhang et al., 2008), *Labor*, *Capital*, and *Energy* consumption of the tourism industry were selected as the input indicators to evaluate the eco-efficiency of China’s tourism industry. Specifically, *Labor* was expressed by the annual number of employees in the tourism industry and *Capital* was represented by the investment on fixed assets of the tourism industry. Additionally, *Energy* was expressed by the energy consumption of the tourism industry, which was calculated by the bottom-up method (Section 3.1). In term of outputs, this paper selected *Tourism Revenue* as the desirable output and *Carbon Emission* as the undesirable output, which was also calculated by the bottom-up method. 

In order to eliminate the influence of environmental effects, this paper selected *Per Capita GDP*, *Consumption Level*, *Urbanization Level*, and *Trade Openness* to conduct the SFA regression. The *Per Capita GDP* is expressed by the ratio of the annual GDP to the total population of each province. The *Consumption Level* is expressed by the annual consumption per person in each province, which was a basis for residents to carry out tourism consumption, and has a decisive effect on the tourism cost and tourism preferences. The *Urbanization Level* is expressed by the ratio of the urban population to the total population of the region and *Trade Openness* is denoted by the percentage of total import and export to GDP. Meanwhile, the adjusted inputs can be obtained by the SFA regression and Equation (8). The descriptive statistics of inputs and outputs are shown in Table 3.

## 4. Empirical Results

### 4.1. Stage Ⅰ: The Comprehensive Eco-Efficiency of China’s Tourism Industry

In Stage Ⅰ, the undesirable SBM model was employed to measure the comprehensive eco-efficiency of China’s provincial tourism industry from 2008 to 2017 (Table 4). 

As shown in Table 4, Tianjin and Jiangsu are superior to other provinces from 2008 to 2017, because their eco-efficiency value is equal to 1, indicating the frontier is conducted by these provinces, while the eco-efficiency values of Qinghai, Ningxia, and Xinjiang are 0.23, 0.19, and 0.27 respectively, which are significantly lower than other provinces. In addition, 11 provinces did not have good ecology performances; their average eco-efficiency value is less than 0.5. The eco-efficiency of most provinces has experienced an obvious increase during the past ten years, with the average eco-efficiencies increasing from 0.47 (2008) to 0.69 (2017). Finally, it can be found that the tourism industry in developed provinces has a better ecology performance than in less-developed provinces, demonstrating that the comprehensive eco-efficiency of China’s tourism industry has potential for improvement, especially in less-developed provinces. 

**Figure 2:**
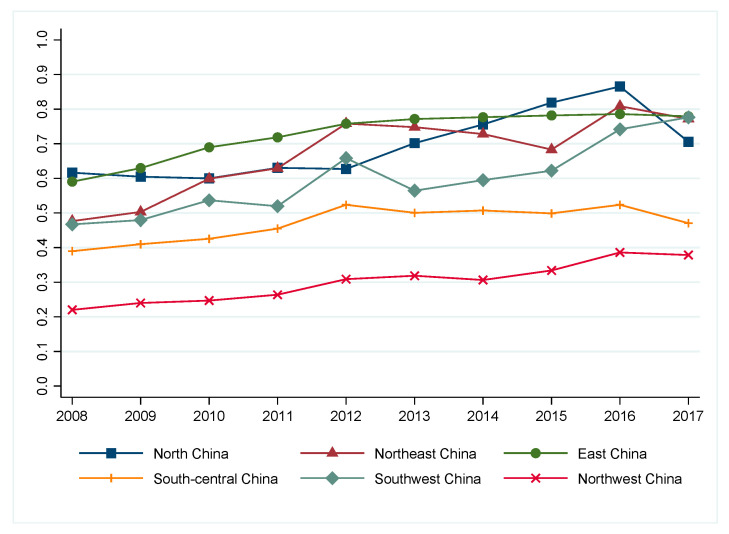
Regional comprehensive eco-efficiency of China’s tourism industry (2008–2017).

Figure 2 shows the trend of the regional average eco-efficiency from 2008 to 2017. It can be seen that the eco-efficiency in East China, Northeast China, North China, and Southwest China was higher than in South-Central China and Northwest China. In addition, there was an obvious fluctuating increase in North China, Northeast China, and Southwest China from 2008 to 2017, while East China, Northwest China, and South-Central China did not illustrate an apparent decreasing or increasing trend. The eco-efficiency in Northeast China and South-Central China witnessed a significant gap compared with other regions, which was puzzling for their rich natural and human tourism resources.

### 4.2. Stage Ⅱ: The Influences of Exterior Environmental Factors on Eco-Efficiency

This paper then established an SFA model with four selected environmental variables as explanatory variables and the slacks of inputs in Stage Ⅰ as the explained variables. Then, the original inputs could be adjusted according to the regression coefficient, thereby eliminating the influence of certain external factors and making all DMUs face fair external conditions and luck. The results of the SFA model are shown in Table 5.

It is illustrated in Table 5 that γ is significant and close to 1, indicating that the impact of external environment dominated the inefficiency in tourism industry. In addition, almost all the selected environmental variables had significant influence on the input slacks, meaning that the environmental variables had an impact on the difference between the actual inputs and the potential optimal inputs to achieve the same output and indicating that the external environment will affect the efficiency value of each DMU. Hence, it is necessary to eliminate the influence of external factor variables.

*Per Capita GDP* has a significant negative impact on each input slack, indicating that the development of the economy narrowed the difference between the actual inputs and the potential optimal inputs; hence, each DMU needs fewer inputs to achieve the same outputs, thus promoting the improvement of the eco-efficiency in the tourism industry. It was consistent with the fact that developed provinces in the eastern regions tend to have a higher eco-efficiency in Stage Ⅰ. It can be explained that developed provinces pay more attention to environmental protection and these provinces might be on the right side of the environmental Kuznets curve (EKC); the scale effect and agglomeration effect brought by economic development can effectively realize the balanced development of economy and environment [46]. 

*Consumption Level* has a significant positive impact on the slack of capital, but a negative impact on the slack of labor. This result indicates that a higher consumption level will increase the capital inputs in tourism industry while reducing the labor inputs. The substitution between capital and labor makes the impact of consumption on tourism eco-efficiency uncertain. 

*Urbanization Level* has a significant positive impact on the slack of labor and energy, indicating that a higher urbanization level will increase the labor and energy inputs, which demonstrates that the development of urbanization would have a certain negative impact on the eco-efficiency of the tourism industry. This might result from increasing urban population and the agglomeration of resources in the tertiary industry, especially in the tourism industry, which has led to less efficient uses of resources. 

*Trade Openness* has a significant positive impact on the slack of capital, but a negative impact on the slack of labor and energy consumption. The substitution between capital, labor, and energy makes the impact of trade openness on tourism eco-efficiency uncertain. With the development of China’s economy and the level of openness, the tourism demand from home and abroad is increasing, accompanying the rapid growth of capital investment and technology spillover into the tourism industry. On the other hand, the increase in tourist numbers is also putting more pressure on the environment.

### 4.3. Stage Ⅲ: The Environmental-Adjusted Eco-Efficiency of China’s Tourism Industry

The analysis in Stage Ⅱ illustrates that the environmental variables have a certain influence on eco-efficiency. Therefore, this paper employed the adjusted inputs to measure the environmental-adjusted eco-efficiency of China’s tourism industry based on the undesirable SBM model. 

It can be seen from Table 4 and Table 6 that, when all DMUs faced the most unfavorable external environment and luck, the average eco-efficiency of 30 provinces decreased from 0.58 (Stage Ⅰ) to 0.52 (Stage Ⅲ). The eco-efficiency of most provinces (except for Jiangsu, Sichuan, Shandong, Henan, and Guangdong) showed an obvious decrease in the period from 2008 to 2017. In addition, Beijing, Tianjing, Jiangsu, and Liaoning performed better than other provinces from 2008 to 2017, as the average eco-efficiency of these provinces was 0.87, 0.88, 1, and 0.89, respectively, while Ningxia and Qinghai were much lower than the other provinces. In addition, the eco-efficiency of 14 provinces (including Hebei, Inner Mongolia, Heilongjiang, Jilin, Hunan, Guangxi, Hainan, Chongqing, Yunnan, Shaanxi, Ningxia, Gansu, Qinghai, and Xinjiang) was less than 0.5, indicating that, whether eliminating the influence of external environment or not, the tourism industry in developed provinces generally has a better low-carbon performance than in less-developed provinces.

**Figure 3:**
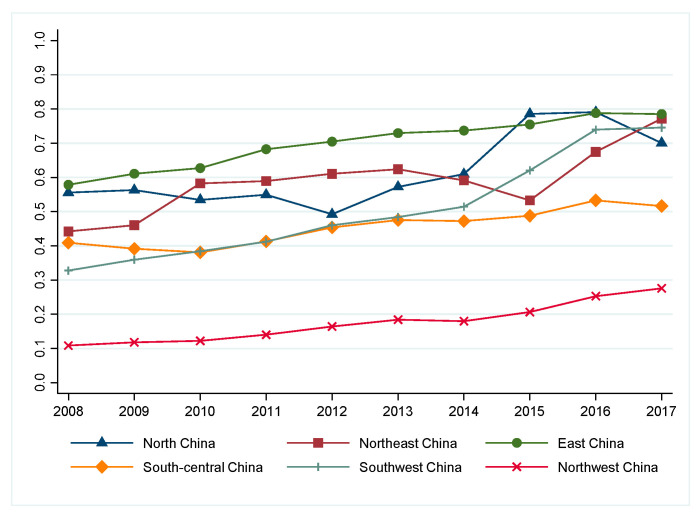
Regional real eco-efficiency of China’s tourism industry (2008–2017).

Figure 3 shows the regional trend of the average eco-efficiency of the tourism industry in Stage Ⅲ. It can be seen that the tourism eco-efficiency in Northwest China and South-Central China is relatively lower than other regions and the regional gaps still exist in China due to economic and social development. There was a slight increasing tendency in real eco-efficiency value in East China, Southwest China, and Northwest China, while South-Central China, Northeast China, and North China presented an obvious fluctuating increasing tendency. However, South-Central China has narrowed the gap with other regions, while Northwest China still has a depression in the low-carbon performance of the tourism industry.

**Figure 4:**
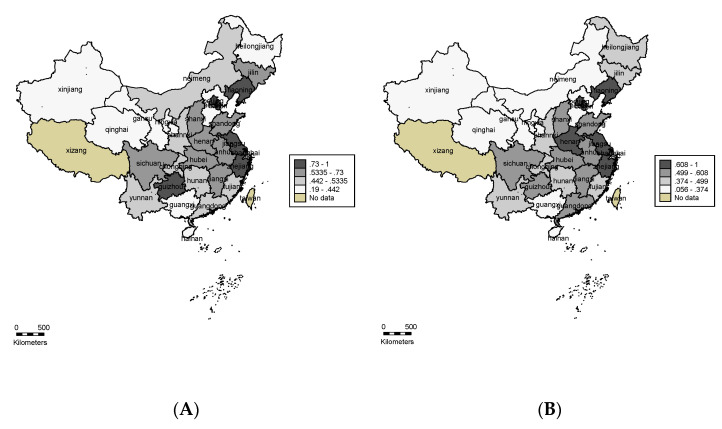
Spatial distribution of eco-efficiency in China’s tourism industry. (**A**) Comprehensive eco-efficiency of the tourism industry. (**B**) Adjusted eco-efficiency of the tourism industry.

The average eco-efficiency in Stage Ⅲ and Stage Ⅰ of each province is represented in Figure 4 to investigate the spatial distribution and regional gaps of China’s tourism industry. As illustrated in Figure 4, the eco-efficiency of the tourism industry in the whole country shows a decline after eliminating the environmental value and statistical error. In addition, the value of eco-efficiency shows a significant trend of regional agglomeration and shows a decreasing trend from Eastern to Western regions in both Stage Ⅲ and Stage Ⅰ, indicating that disparity among the east, middle, and west areas still exists.

In particular, after the adjustment of environmental factors and statistical noise, the tourism eco-efficiency in midland China shows a significant decline when facing the worst external environment and luck. This phenomenon shows that, although these provinces have relatively rich tourism resources and excellent natural environments (such as Guizhou, Heinan, and Inner Mongolia), the state of development of their tourism industry and the efficiency of their internal management greatly limit the development of these advantages. On the contrary, the coastal provinces are not as rich in tourism resources as the mainland provinces and their efficient management levels have enabled their tourism industries to show high eco-efficiency.

## 5. Discussion 

### 5.1. Decomposition of Tourism Eco-Efficiency

**Figure 5:**
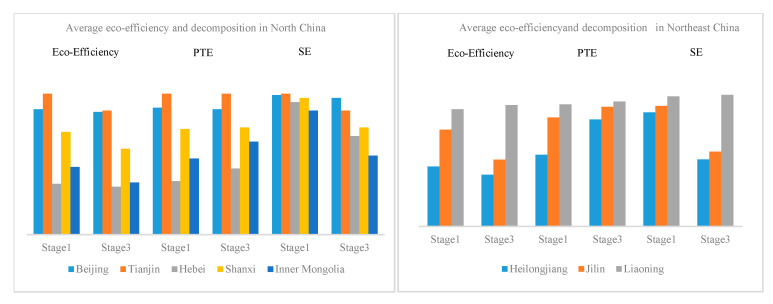

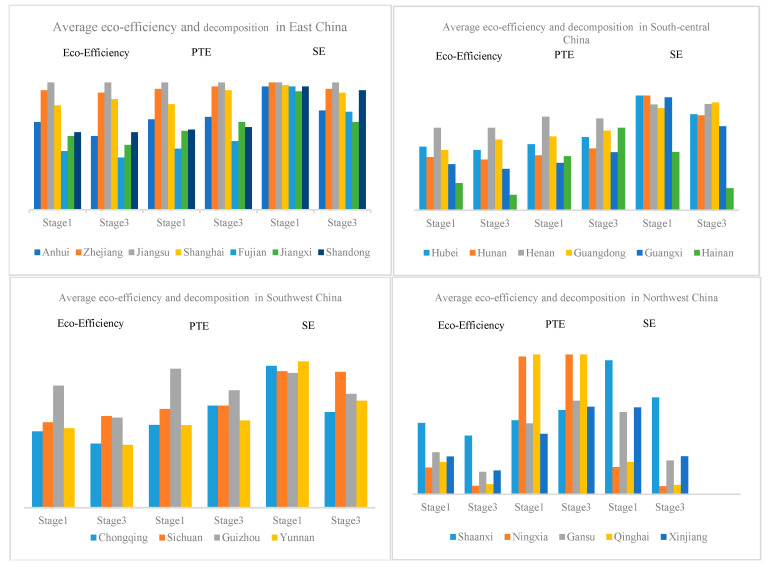
The average eco-efficiency and its decomposition of China’s tourism industry.

Then, the eco-efficiency of China’s tourism industry was decomposed into pure technical efficiency (PTE) and scale efficiency (SE) according to the Luenberger index, to uncover the intrinsic drives behind the eco-efficiency value. PTE expressed the efficiency of existing production technology without considering economies of scale, while SE indicated the overall efficiency change due to the scale effect. Figure 5 shows the results.

In North China, the eco-efficiency of the tourism industry in Tianjin was always the highest, showing that the eco-efficiency of the tourism industry in Tianjin had been in an effective state and is in a leading position in North China. Beijing’s eco-efficiency of the tourism industry was also slightly lower than Tianjin’s. Regardless of whether the external environmental factors are considered, the eco-efficiency value of Hebei province was the lowest in North China. This is mainly because the PTE of Hebei was far lower than other provinces; only 0.38 in Stage Ⅰ and 0.47 in Stage Ⅲ. 

In Northeast China, the situation was quite different. Whether in Stage Ⅰ or Stage Ⅲ, Liaoning had the highest eco-efficiency value, while Heilongjiang performed the worst. After considering the external environmental factors, there was a significant decrease in TE, which was mainly caused by the decrease in SE. Among them, Heilongjiang experienced a dramatic decrease in SE, from 0.84 to 0.49. These results indicated that, compared with the level of technology in the tourism industry, the adjustment of the external environment limited the scale effect of tourism, which contributed to the inefficiency of internal operations and management for tourism activities.

In East China, the eco-efficiency of the tourism industry varied greatly among different provinces. In Stage Ⅰ, Jiangsu had the highest average travel eco-efficiency score (1.00), followed by Zhejiang (0.94), and Jiangxi with the lowest (0.41). After the adjustment of environmental factors, the eco-efficiency of the tourism industry still followed this order, indicating that Jiangsu had been in an effective state and was in a leading position in East China. It illustrated that, after the adjustment, there were no significant changes in eco-efficiency and its decomposition, indicating the management level in the tourism industry in East China was excellent. 

In South-Central China, the eco-efficiency of the tourism industry was not high, whether or not the external environmental factors were considered, especially in Guangxi and Hainan. However, behind the poor low-carbon performance of these provinces, the SE was pretty high, which means the technology level limits the scale effects of the tourism industry in these provinces. The potential for the sustainable development of the tourism industry in this region relies on the improvement of internal management levels that are brought about by technology innovation in the tourism industry. 

In Southwest China, the eco-efficiency of Guizhou was relatively high in Stage Ⅰ and experienced a slight decrease after excluding the influence of environmental factors; this also occurred in other provinces of the southwest. The increase or decrease in the eco-efficiency and its decomposition of the tourism industry in these provinces was small, indicating the environmental factors in this region were not dominant in tourism eco-efficiency and the management level inside the tourism industry was the direct driver of its low-carbon performance.

Northwest China is a region traditionally rich in tourism resources but less economically developed, which was demonstrated by its lower tourism eco-efficiency in both Stage Ⅰ and Stage Ⅲ. The low TE efficiency, as well as the scale effect, was the main reason for this situation; it does not change with the adjustment of environmental factors. These results indicate that there is great potential for the promotion of tourism development and low-carbon performance in this region.

### 5.2. Quadrant Analysis

**Figure 6:**
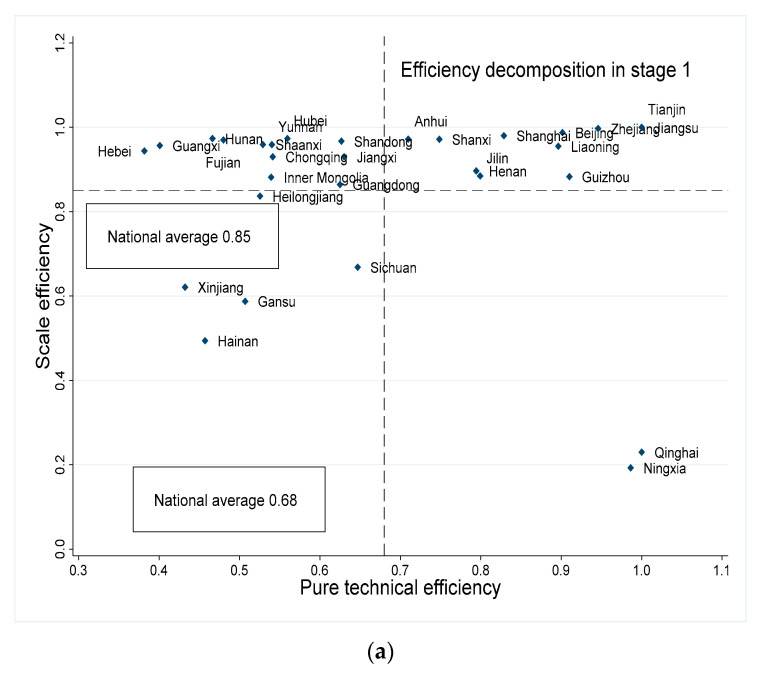

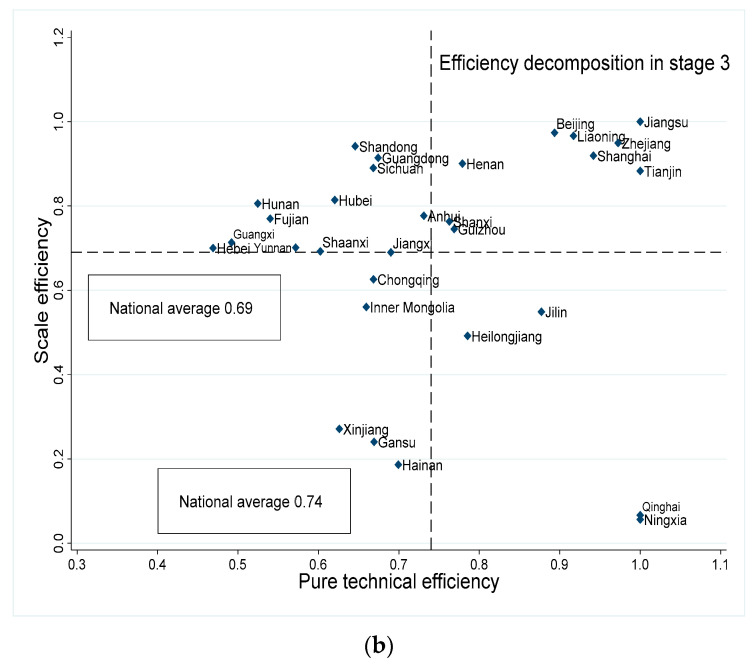
Categorization of PTE and SE of 30 provinces. (**a**) PTE and SE in Stage 1; (**b**) PTE and SE in Stage 3.

On the basis of the above analysis about eco-efficiency and its decomposition characteristics of each region and province, this section further discusses the categorization of the PTE and SE of eco-efficiency in the first and third stages, to provide policy implications for sustainable tourism development towards different types of provinces. According to the average values of PTE and SE, 30 provinces were grouped into four categories, namely, high–high group, high–low group, low–high group, and low–low group (Figure 6). 

In Stage Ⅰ, there were 11 provinces in the high–high group, since all provinces were faced with the worst external environment and luck after adjustment; provinces in the high–high group decreased to 9 in Stage Ⅲ. Regardless of whether considering the exterior environmental factors, Beijing, Tianjin, Shanxi, Liaoning, Zhejiang, Jiangsu, Shanghai, Henan, and Guizhou were all categorized into the high–high group, indicating that the excellent technological and management level, as well as the scale effect in the tourism industry, enabled these provinces to overcome the negative impact of the unfavorable external environment and to achieve sustainable development in the tourism industry. On the contrary, Anhui and Jilin fell into the low–high and high–low groups, indicating that the excellent external environment pulled up their PTE and SE and that they need to make improvements in upgrading tourism technology and exploiting the economies of scale in tourism in order to fully exploit their advantages in the external environment. 

The high–low group consisted of the provinces with high PTE and low SE. Regardless of whether considering the exterior environmental factors, Ningxia and Qinghai were all categorized into the low–high group. The SEs of Ningxia and Qinghai were 0.06 and 0.07, respectively, which is far lower than in other provinces, meaning that these regions need to focus on improving scale efficiency and realizing the centralized allocation of resources. In addition, Heilongjiang entered into the high–low group from the low–low group, indicating that the external environment limited the effectiveness of its PTE in promoting eco-efficiency.

The low–high group is composed of the provinces with a lower PTE and a higher SE than the national average level. It is illustrated that Anhui, Fujian, Guangdong, Guangxi, Hebei, Hubei, Hunan, Jiangxi, Shaanxi, Shandong, and Yunnan are all categorized into the low–high group. The PTEs of these provinces are all smaller than the national average level. Therefore, this kind of province should focus on the improvement of PTEs in future development, that is, improving the technical management level in production and operation activities.

The low–low group consisted of the provinces with lower PTEs and SEs than the national average level. Hainan, Gansu, and Xinjiang fell into the low–low group; they are natively rich in tourism resources. Although there is no shortage of tourism resources in these areas, lack of education, poor technology, and weak management will undoubtedly make the use of resources inefficient. This inefficiency is not only reflected in the technology gaps but also in the limited scale effect of its tourism industry. Interestingly, Hainan, one of the most popular tourist destinations in China or even Southeast Asia, presented a poor tourism ecological performance, which is not consistent with its status. The lower PTE and SE show that, in Hainan Island, the rich tourism resources and tourism development experiences have not brought the improvement of tourism technology efficiency and its scale effect has not been reflected. It can be explained by the unbalanced development in Hainan, where most of the investments and visitors are gathering in cities such as Sanya and Haikou and the spillover effect and the knowledge innovation diffusion are limited by the investments in tourism and infrastructure [47]. As a result, tourism development in other cities such as Tunchang and Ledong are still in a relatively backward stage, restricting the promotion of Hainan’s overall tourism performance [48].

## 6. Conclusions, Policy Implication, and Further Research

Tourism is an important part of the tertiary industry and an overwhelming driver of national economic development. As an industry that directly faces natural ecology, the ecological and environmental issues in the development of the tourism industry deserve more attention. This paper combined the undesirable SBM model to measure the eco-efficiency of the tourism industry in China’s 30 provinces from 2008 to 2017 and discussed the reasons for its inefficiency and the paths of sustainable tourism development in different categories of provinces. The main findings of this paper can be summarized as follows. 

Firstly, the tourism eco-efficiency of most provinces has experienced an obvious increasing tendency during the past ten years, while the tourism industry in the developed provinces generally has a better ecology performance than in the less-developed provinces. The comparison of the before-and-after environmental adjustment indicated that the efficiency of the internal management in the tourism industry greatly limited the advantage of tourism resources in South-Central and Northwest China. 

Secondly, the decomposition of tourism eco-efficiency shows that the contribution of TE and SE to tourism eco-performance varies across different regions. Specifically, SE was the direct driver of the poor low-carbon performance in Northeast China, while PTE dominated the tourism eco-efficiency in South-Central China. These two issues have together led to the lack of utilization of the rich tourism resources and natural environment in Southwest China.

Thirdly, the quadrant analysis before and after the environmental adjustment provided different paths for the promotion of tourism low-carbon performances in different categories of provinces. The high–low groups should focus on unleashing the scale potential of the tourism industry, while the low–high group needs to improve tourism technology. The low–low group needs to promote the low-carbon development of tourism by simultaneously leveraging the scale effect and upgrading technology. 

By shedding light on the measurement and decomposition of eco-efficiency in the tourism industry, this paper provided the following policy implication to promote the sustainable development of China’s tourism industry:

(1) Reducing regional differences is one of the keys to improving the eco-efficiency of China’s tourism industry. The eco-efficiency of the tourism industry in Northwest China is at the lowest level in the country, but, in fact, those provinces with rich natural resources may not have been fully planned and utilized. This is one way to improve tourism efficiency in the Northwest region by developing and utilizing its rich tourism resources as much as possible and by attracting tourists to increase its income. In addition, from the regional classification based on PTE and SE, the region located in the high–high group should take full use of its economic and social location advantages, further enhancing its eco-efficiency of the tourism industry. Other regions should take appropriate measures to improve scale efficiency or pure eco-efficiency. 

(2) The steady development of the economy has laid a foundation for the sustainable development of the tourism industry. Regions with strong economic strength could provide better infrastructure and technical support for tourism development. According to the empirical results of this paper, the eco-efficiency of the tourism industry in economically developed provinces is higher than in less-developed provinces. Its tourism industry is also relatively sustainable. Thus, it is necessary to first improve the regional economic level before the development of the tourism industry.

This paper discusses eco-efficiency and its driving force in China’s tourism industry. Since the traditional DEA model is a black box that pays less attention to the specific relationship between different inputs and outputs, as well as the interaction within the different production processes, further research may combine the undated network-DEA or process-DEA to conduct a more micro-analysis. Additionally, with the global pandemic of COVID-19, the tourism industry has experienced unprecedented changes in patterns and habits of transportation, accommodation, and tourism activities. The impact of COVID-19 on tourism eco-efficiency and its driving force needs further research.

## Figures and Tables

**Table 1:** Energy consumption and carbon emissions of tourism-related transport.

Transportation	Railway	Highway	Water Transport	Civil Aviation
Energy consumption (MJ/passenger)	1	1.8	2	12
CO_2_ emission (g/passenger)	27	133	106	137

**Table 2:** Energy consumption and carbon emissions of tourism activities.

Tourism Intention	Sightseeing	Leisure Vacation	Business Meetings	Visiting Friends	Others
Energy consumption (MJ/person)	8.5	26.5	16	12	3.5
CO_2_ emission (g/person)	417	1670	786	591	172

**Table 3:** Descriptive statistics of main variables.

Variable	Type	Unit	OBS	Min	Max	Mean	SD
*Labor*	Input	10,000	300	0.69	32.71	7.33	5.4
*Capital*	Input	CNY 100 million	300	77.26	6280.4	1645.34	1189.95
*Energy*	Input	100 million MJ	300	12.41	1172.42	233.79	200.68
*Tourism Revenue*	Desirable Output	CNY 100 million	300	40.53	11,662.18	2691.94	2286.49
*Carbon Emission*	Undesirable Output	10,000 ton	300	17.51	2204	405.5	391.21
*Per Capita GDP*	Environmental Variables	CNY	300	44,581.79	128,994.18	8824	23,778.74
*Consumption Level*		CNY	300	15,644.71	53617	4426	8812.26
*Urbanization Level*		%	300	0.29	0.895	0.547	0.133
*Trade Openness*		%	300	0.006	1.671	0.301	0.363
*Adjust Labor*	Adjusted Inputs	10,000	300	0.67	39.88	6.97	5.11
*Adjust Capital*		CNY 100 million	300	74.18	5870.26	1347.18	997.56
*Adjust Energy*		100 million MJ	300	11.98	1077.81	203.67	196.51

**Table 4:** Comprehensive eco-efficiency of China’s tourism industry (2008–2017).

Region	Province	2008	2009	2010	2011	2012	2013	2014	2015	2016	2017	Average
North	Beijing	1.00	1.00	0.80	1.00	0.74	1.00	0.92	1.00	1.00	0.45	0.89
China	Tianjin	1.00	1.00	1.00	1.00	1.00	1.00	1.00	1.00	1.00	1.00	1.00
	Hebei	0.20	0.20	0.31	0.27	0.33	0.34	0.40	0.50	0.63	0.45	0.36
	Shanxi	0.53	0.45	0.52	0.51	0.61	0.72	0.96	1.00	1.00	1.00	0.73
	Inner Mongolia	0.36	0.37	0.37	0.37	0.45	0.45	0.49	0.59	0.70	0.63	0.48
Northeast	Heilongjiang	0.44	0.41	0.48	0.51	0.61	0.48	0.36	0.38	0.43	0.32	0.44
China	Jilin	0.42	0.46	0.48	0.53	0.66	0.77	0.83	1.00	1.00	1.00	0.71
	Liaoning	0.57	0.64	0.83	0.85	1.00	1.00	1.00	0.67	1.00	1.00	0.86
East	Anhui	0.44	0.46	0.47	0.66	0.78	0.76	0.72	0.79	0.89	0.93	0.69
China	Zhejiang	0.68	0.76	1.00	1.00	1.00	1.00	1.00	1.00	1.00	1.00	0.94
	Jiangsu	1.00	1.00	1.00	1.00	1.00	1.00	1.00	1.00	1.00	1.00	1.00
	Shanghai	0.63	0.76	1.00	1.00	1.00	1.00	1.00	0.82	0.52	0.43	0.82
	Fujian	0.53	0.50	0.44	0.42	0.41	0.44	0.45	0.45	0.46	0.52	0.46
	Jiangxi	0.36	0.36	0.39	0.41	0.50	0.54	0.61	0.71	0.91	1.00	0.58
	Shandong	0.50	0.57	0.53	0.54	0.62	0.66	0.65	0.70	0.71	0.59	0.61
South-Central	Hubei	0.32	0.37	0.47	0.51	0.65	0.66	0.64	0.63	0.63	0.58	0.54
China	Hunan	0.34	0.34	0.42	0.38	0.48	0.50	0.48	0.51	0.58	0.50	0.45
	Henan	0.74	0.77	0.64	0.67	0.77	0.73	0.73	0.70	0.67	0.63	0.70
	Guangdong	0.46	0.45	0.48	0.51	0.57	0.51	0.55	0.56	0.54	0.46	0.51
	Guangxi	0.24	0.28	0.32	0.37	0.43	0.39	0.43	0.38	0.53	0.50	0.39
	Hainan	0.24	0.24	0.24	0.28	0.24	0.21	0.22	0.22	0.19	0.17	0.23
Southwest	Chongqing	0.42	0.43	0.49	0.51	0.56	0.53	0.54	0.54	0.48	0.53	0.50
China	Sichuan	0.43	0.41	0.50	0.48	0.60	0.54	0.60	0.66	0.82	0.61	0.56
	Guizhou	0.64	0.68	0.76	0.71	1.00	0.73	0.74	0.74	1.00	0.97	0.80
	Yunnan	0.38	0.40	0.40	0.38	0.48	0.46	0.50	0.55	0.66	1.00	0.52
Northwest	Shaanxi	0.36	0.40	0.41	0.41	0.53	0.56	0.55	0.55	0.66	0.66	0.51
China	Ningxia	0.16	0.19	0.18	0.17	0.19	0.20	0.18	0.19	0.20	0.24	0.19
	Gansu	0.19	0.24	0.23	0.25	0.29	0.32	0.32	0.35	0.37	0.46	0.30
	Qinghai	0.19	0.22	0.19	0.21	0.24	0.24	0.24	0.26	0.29	0.21	0.23
	Xinjiang	0.20	0.16	0.22	0.27	0.29	0.28	0.24	0.32	0.41	0.33	0.27

**Table 5:** The results of the SFA model.

Explanatory Variables	Slack of Labor	Slack of Capital	Slack of Energy
Constant	−63,266.75 ***	−60.04	−766.83 ***
*Per Capita GDP*	−5659.28 ***	−92.42 ***	−47.01 *
*Consumption Level*	−5019.91 ***	312.87 ***	−52.37
*Urbanization Level*	2230.06 ***	−1.22	19.94 ***
*Trade Openness*	−58,994.08 ***	295.89 ***	−84.91 *
γ	0.73 ***	0.83 ***	0.89 ***
LR	143.31	253.21	322.93

Note: ***, and * represent 1%, and 10% significance, respectively.

**Table 6:** Real eco-efficiency of China’s tourism industry (2008–2017).

Region	Province	2008	2009	2010	2011	2012	2013	2014	2015	2016	2017	Average
North	Beijing	1.00	1.00	0.75	0.84	0.71	0.89	0.90	1.00	1.00	0.59	0.87
China	Tianjin	1.00	1.00	1.00	1.00	0.72	0.78	0.74	1.00	0.78	0.81	0.88
	Hebei	0.17	0.18	0.26	0.24	0.28	0.30	0.35	0.47	0.63	0.52	0.34
	Shanxi	0.32	0.32	0.37	0.38	0.46	0.55	0.68	1.00	1.00	1.00	0.61
	Inner Mongolia	0.28	0.31	0.28	0.28	0.30	0.33	0.37	0.45	0.55	0.59	0.37
Northeast	Heilongjiang	0.39	0.39	0.41	0.42	0.44	0.42	0.29	0.35	0.38	0.32	0.38
China	Jilin	0.32	0.35	0.33	0.35	0.39	0.46	0.49	0.59	0.65	1.00	0.49
	Liaoning	0.62	0.64	1.00	1.00	1.00	1.00	1.00	0.66	1.00	1.00	0.89
East	Anhui	0.35	0.38	0.36	0.53	0.63	0.64	0.61	0.68	0.79	0.81	0.58
China	Zhejiang	0.67	0.74	0.83	1.00	1.00	1.00	1.00	1.00	1.00	1.00	0.92
	Jiangsu	1.00	1.00	1.00	1.00	1.00	1.00	1.00	1.00	1.00	1.00	1.00
	Shanghai	0.80	0.85	1.00	1.00	1.00	1.00	1.00	0.84	0.65	0.55	0.87
	Fujian	0.45	0.43	0.37	0.37	0.35	0.38	0.40	0.41	0.45	0.53	0.41
	Jiangxi	0.25	0.27	0.29	0.32	0.36	0.45	0.52	0.67	0.92	1.00	0.51
	Shandong	0.53	0.60	0.54	0.56	0.59	0.63	0.63	0.68	0.71	0.61	0.61
South-Central	Hubei	0.28	0.34	0.42	0.49	0.57	0.62	0.58	0.59	0.62	0.60	0.51
China	Hunan	0.30	0.33	0.37	0.36	0.41	0.46	0.43	0.48	0.57	0.57	0.43
	Henan	1.00	0.81	0.58	0.62	0.66	0.68	0.67	0.68	0.67	0.66	0.70
	Guangdong	0.56	0.53	0.54	0.58	0.62	0.60	0.61	0.66	0.67	0.59	0.60
	Guangxi	0.19	0.23	0.26	0.31	0.34	0.37	0.40	0.38	0.52	0.53	0.35
	Hainan	0.11	0.11	0.12	0.13	0.13	0.13	0.13	0.15	0.14	0.15	0.13
Southwest	Chongqing	0.32	0.34	0.37	0.40	0.42	0.44	0.44	0.48	0.45	0.51	0.42
China	Sichuan	0.38	0.41	0.46	0.47	0.54	0.56	0.62	0.87	1.00	0.70	0.60
	Guizhou	0.35	0.39	0.42	0.47	0.53	0.57	0.59	0.66	0.90	1.00	0.59
	Yunnan	0.25	0.29	0.29	0.30	0.36	0.36	0.41	0.48	0.61	0.77	0.41
Northwest	Shaanxi	0.29	0.32	0.31	0.34	0.41	0.45	0.45	0.46	0.57	0.61	0.42
China	Ningxia	0.03	0.04	0.04	0.05	0.05	0.06	0.06	0.06	0.08	0.09	0.06
	Gansu	0.07	0.09	0.10	0.12	0.14	0.17	0.18	0.21	0.23	0.31	0.16
	Qinghai	0.04	0.05	0.04	0.05	0.06	0.07	0.07	0.09	0.10	0.10	0.07
	Xinjiang	0.10	0.08	0.11	0.14	0.16	0.17	0.15	0.21	0.29	0.27	0.17

## Data Availability

The datasets used in this study are available from the corresponding author on reasonable request.
